# Reconstruction and analysis of genome-scale metabolic model of weak Crabtree positive yeast *Lachancea kluyveri*

**DOI:** 10.1038/s41598-020-73253-3

**Published:** 2020-10-01

**Authors:** Piyush Nanda, Pradipta Patra, Manali Das, Amit Ghosh

**Affiliations:** 1 Department of Biotechnology, Indian Institute of Technology Kharagpur, Kharagpur, West Bengal 721302 India; 2 School of Bioscience, Indian Institute of Technology Kharagpur, Kharagpur, West Bengal 721302 India; 3 School of Energy Science and Engineering, Indian Institute of Technology Kharagpur, Kharagpur, West Bengal 721302 India; 4 P.K. Sinha Centre for Bioenergy and Renewables, Indian Institute of Technology Kharagpur, Kharagpur, West Bengal 721302 India

**Keywords:** Computational biology and bioinformatics, Systems biology

## Abstract

*Lachancea kluyveri*, a weak Crabtree positive yeast, has been extensively studied for its unique URC pyrimidine catabolism pathway. It produces more biomass than *Saccharomyces cerevisiae* due to the underlying weak Crabtree effect and resorts to fermentation only in oxygen limiting conditions that renders it as a suitable industrial host. The yeast also produces ethyl acetate as a major overflow metabolite in aerobic conditions. Here, we report the first genome-scale metabolic model, iPN730, of *L. kluyveri* comprising of 1235 reactions, 1179 metabolites, and 730 genes distributed in 8 compartments. The in silico viability in different media conditions and the growth characteristics in various carbon sources show good agreement with experimental data. Dynamic flux balance analysis describes the growth dynamics, substrate utilization and product formation kinetics in various oxygen-limited conditions. We have also demonstrated the effect of switching carbon sources on the production of ethyl acetate under varying oxygen uptake rates. A phenotypic phase plane analysis described the energetic cost penalty of ethyl acetate and ethanol production on the specific growth rate of *L. kluyveri*. We generated the context specific models of *L. kluyveri* growing on uracil or ammonium salts as the sole nitrogen source. Differential flux calculated using flux variability analysis helped us in highlighting pathways like purine, histidine, riboflavin and pyrimidine metabolism associated with uracil degradation. The genome-scale metabolic construction of *L. kluyveri* will provide a better understanding of metabolism behind ethyl acetate production as well as uracil catabolism (pyrimidine degradation) pathway. iPN730 is an addition to genome-scale metabolic models of non-conventional yeasts that will facilitate system-wide omics analysis to understand fungal metabolic diversity.

## Introduction

*Lachancea kluyveri*, previously known as *Saccharomyces kluyveri*, is a weak Crabtree positive yeast that has been presented in numerous studies due to its quintessential metabolic properties^1–3^. Its unique pyrimidine catabolism pathway has been well characterized and its importance has been discussed^1,4^. It has the ability to utilize purines and pyrimidines as sole nitrogen sources which makes it a great interest to the pharmaceutical industry^5^. The ability to assimilate and ferment melibiose by *L. kluyveri* is also a differentiating feature from other characterized yeasts^3^. The organism diverged from *Saccharomyces cerevisiae* after the Whole Genome Duplication (WGD) event^4^. Previously, using searchDOGS algorithm, the position of the whole genome duplication event has been described for 11 yeast species and they marked the location of the whole genome duplication (WGD) event which separated the *Saccharomyces* genus from *Lachancea*^6^. The effect of the WGD event is reflected in the number of ortholog duplicates of *L. kluyveri* genes in *S. cerevisiae.* The single knockout simulation would also explain the effect of such duplicate genes on the viability of the model and metabolic flux. Genome-scale metabolic models (GEMs) are one of the important systems biology tools as they integrate genomic information, transcriptomics, metabolomics and associated experimental data for a given organism into a whole-cell metabolic network^7–9^. Moreover, complementary to general omics analysis, GEMs provide a scope to integrate ‘omics’ data into metabolic networks and help to predict condition-specific metabolic capabilities. To date, GEMs have been successfully applied to design microbial strains for bioproduction and evaluate their capabilities in different conditions^10^. Specifically, the model yeast *S. cerevisiae* was the first choice among the eukaryotic organisms to be fully sequenced^11,12^, and it is one of the conventional workhorses in cell factory engineering for bio-production of several compounds with various applications in chemical^13,14^, food^15,16^ and pharmaceutical industries^17^. These models are widely being used for various biotechnological predictions and experimental designs. However, non-conventional yeast species include important human pathogens or have also been reported to be suitable platforms for several biotechnological applications and various models have been therefore reconstructed for many yeasts.

The weak Crabtree energy metabolism of *L. kluyveri* allows minimal fermentation in aerobic conditions^18^. Crabtree effect describes the preferential respiro-fermentative metabolism of yeasts i.e. production of ethanol in aerobic condition over respiratory metabolism. Since *L. kluyveri* is a weak Crabtree-positive, this enables it to produce more biomass compared to *S. cerevisiae*, which diverts substantial flux towards ethanol production even in aerobic conditions^2^. This makes it a lucrative host for protein production^19^. Ethyl acetate has also been measured to be a major overflow metabolite in growth studies^2^. *L. kluyveri*, therefore, could be leveraged to uncover the mechanisms behind these metabolic routes and also serves as a potential industrial organism.

When GEMs are combined with constraint-based algorithms, they offer an excellent chance to analyze metabolism and genotype–phenotype relationships^10^. Genome-scale metabolic reconstructions (GEM) are expanding our understanding of cellular metabolism and its dynamics. It supplies us with a systems-level picture of the metabolism derived from its genotype. GEMs have been used to analyze disease phenotypes and improvise the production of chemicals from cell factories. Fungal GEMs have been widely used for metabolic engineering efforts to overproduce target metabolites like fuels and chemicals^13,14,20,21^. As an obvious target, *S. cerevisiae*’s GEM was constructed iteratively due to its immense industrial and academic importance. Beginning from iFF708i^9^, other metabolic models like iND750^22^, iMH805/775^23^ and iMM904^24^ have been reconstructed. iMH805 has gene regulatory information making it a powerful model to analyze the coordination of metabolic pathways with gene circuits^23^. This has begun the quest for exploring related yeast strains that can furnish novel phenotypes. In this regard, numerous yeast GEMs have been published for *Kluyveromyces lactis*^25^, *Pichia pastoris*^26^ and *Yarrowia lipolytica*^27^. GEMs can be used to visualize a systems-level effect of environmental conditions and induced perturbations.

To understand the metabolic capabilities of *L. kluyveri*, here we built the first GEM named iPN730, which comprises of 1235 reactions, 1179 metabolites, 730 genes, and 8 compartments. *Lachancea kluyveri* NRRL-12651 strain was selected for the genome annotation due to the extensive experimental analysis that has been conducted on this organism particularly with respect to ethyl acetate production and uracil (pyrimidine) degradation. Single knockout analysis using Flux Balance Analysis (FBA)^8^ led to a significant agreement with experimental knockout data^28^ for *S. cerevisiae* in various in silico media. Dynamic Flux Balance Analysis (DFBA)^29^ resulted in growth dynamics, product formation that agreed with experimental observation as reported in previous studies. The reported pathways for pyrimidine catabolism and ethyl acetate production are also incorporated into the model to enable further studies. Furthermore, integration of gene expression data into iPN730 for growth on uracil or ammonium salt as the sole nitrogen source helped us in finding pathways associated with uracil degradation. To our knowledge, it is the first attempt to develop a GEM for *L. kluyveri* through which we anticipate a better understanding of the metabolic routes in the organism and streamlining its industrial applications.

## Results

### Genome-scale metabolic reconstruction of *L. kluyveri*

**Figure 1:**
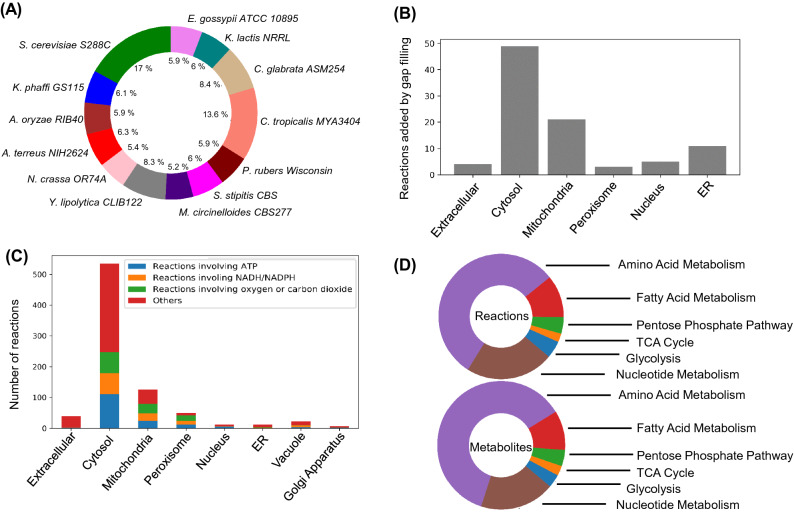
Genome-scale metabolic reconstruction of *Lachancea kluyveri*. (**A**) Distribution of the orthologs of *Lachancea kluyveri* protein coding genes associated with metabolism in various fungi in percentage of total. (**B**) The number of reactions added to the model by manual gap filling with reference to MetaCyc and KEGG contained in various compartments. (**C**) Irreversible reactions in the iPN730 distributed across various compartments. The colour indicates the reactions belonging specific categories involving ATP, NADH/NADPH or metabolic gases like oxygen or carbon dioxide. (**D**) The pathway level subsystem statistics for reactions and metabolites in iPN730.

The genome annotation of *Lachancea kluyveri* NRRL 12,651 (Assembly: ASM14922v1, GenBank assembly accession: GCA_000149225.1) using Yeast Genome Annotation Pipeline^30^ yielded 5,505 ORFs. The draft model from the annotated genome constructed using Build Fungal Model in KBase contained 1180 reactions, 1232 metabolites, and 730 genes. The draft model harbored the integration of data from 13 well-curated fungal genome-scale reconstructions. *S. cerevisiae* was used as the template model due to its high quality and reliability. The homology table generated from the BDBH algorithm for proteome comparison has been incorporated (Table S4, Supplementary Information). This can be used to predict possible duplication, inversion, deletion, insertion and synteny loci between the organisms i.e. *L. kluyveri* and *S. cerevisiae*.The details about the fungal templates used for reconstruction have been described in “Methods” section. In the gene-level data integration statistics obtained from KBase Build fungal model (Fig. 1A), the percentage represented the fraction of protein-coding genes that have an ortholog in a specific fungal template model. *S. cerevisiae* showed the highest similarity (17%) followed by *Candida tropicalis* (13.6%) with *L. kluyveri.*

In a metabolic model, correct mass balance in the reactions is critical for the quality of predictive power^7^**.** Elemental and charge balances can be calculated and analyzed only if the model contains the formula and charge for the metabolites respectively. All the metabolites were assigned formula and charge with reference from CHEBI (Chemical Entities of Biological Interest), PubChem (NIH) and BiGG Database (UCSD)^31^. For metabolites having ambiguous alkyl group (-R), the alkyl group was considered as a conserved moiety “R” in the metabolite formula. The default metabolite ID was replaced by standard nomenclature (MIRIAM) for genome-scale metabolic reconstruction as used elsewhere for well-established models iMM904, iAF1260, etc. The assigned ID captured both the information regarding the chemical name and the compartments of the metabolites. Similar assignment was made for reactions in the reconstruction. Further refinement of the model was necessary with regard to the gaps in the biosynthetic pathways and the ambiguous reversibility of the reactions.

To assign the correct reversibility of the metabolic reactions, the iMM904 model was used as a template and homologous reactions were mapped to the draft model based on modelSEED reaction (Table S5, Supplementary Information). This corrected 82.5% (1018 of 1232 reactions) of the inconsistencies in the reversibility of the reactions. The remaining inconsistencies were resolved by manual curation using COBRApy^32^. Upper and lower bounds for internal reversible reactions were set to a high flux value i.e. 1000 mmol/gdw/h to allow unconstrained flux through the internal metabolic network. For irreversible reactions, a lower flux bound of zero was assigned to restrict any flux in the reverse direction. The reversibility information on such ambiguous reactions was obtained from MetaCyc, KEGG and previously published datasets^33^ on metabolic reactions. The irreversible reactions comprised of reactions that involve: (1) ATP as reactant, (2) Oxygen as the product, and (3) Reactions involving NADH and NADPH. (Fig. 1B). These are due to the high enthalpy of the reactions associated with ATP breakdown, NADH/NADPH consumption and the fact that the cell cannot produce oxygen through its metabolism^7^. Through this semi-automated correction of the reversibility, 879 reactions were subject to irreversible flux bounds.

**Figure 2:**
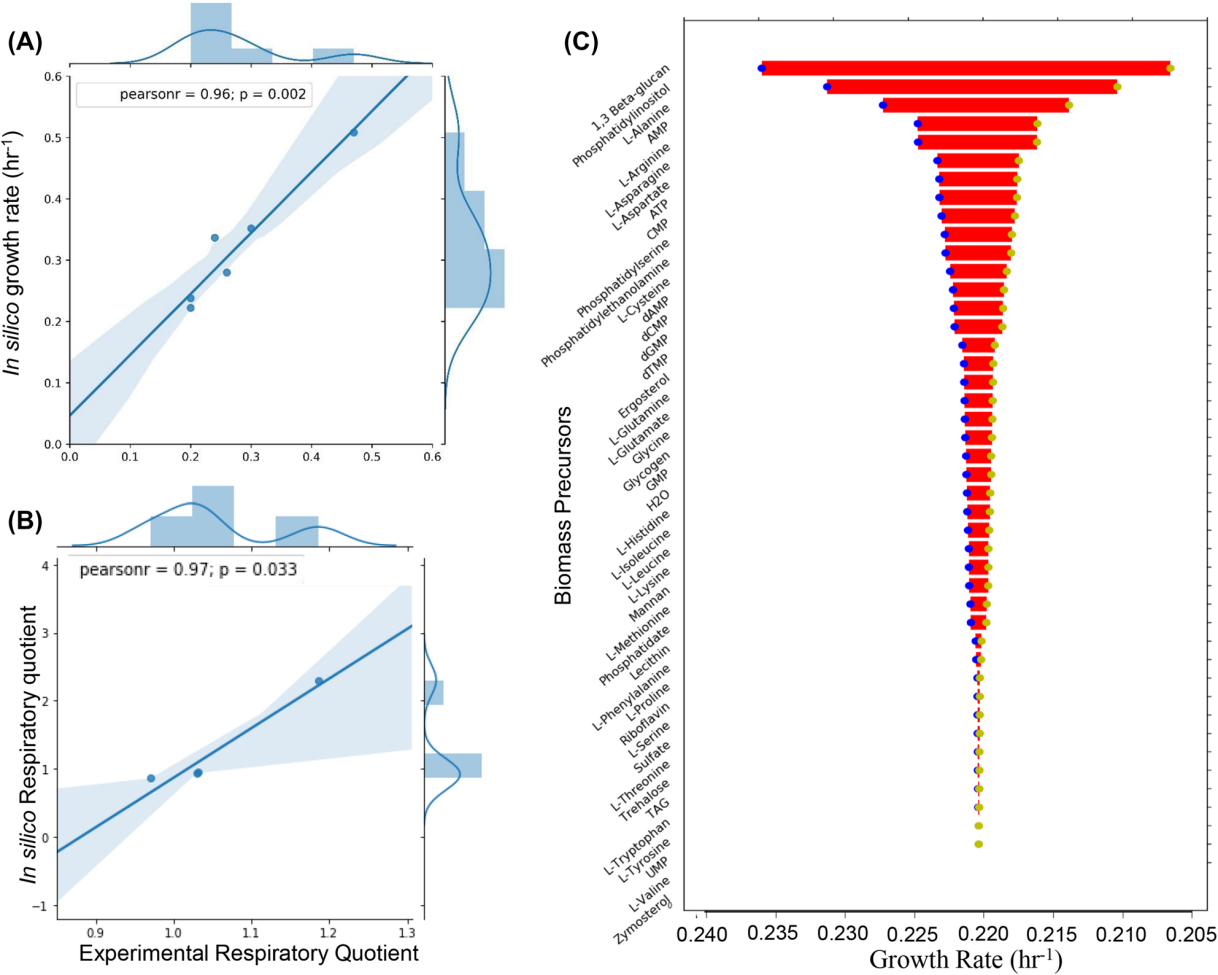
Computational predictions of growth characteristics show good agreement with experimental reports. (**A**) The correlation between the experimental growth rate observed in previous studies and the in silico growth rate from Flux Balance Analysis (FBA) of iPN730. (**B**) The correlation between the experimental respiratory quotient (moles of carbon dioxide released per mole of oxygen consumed) and the in silico values obtained from iPN730. (**C**) The sensitivity analysis of the biomass objective function to variations in the coefficients of biomass precursors in iPN730.

In GEMs, the biomass equation describes the growth of the cells from the biomass precursors like amino acids, carbohydrates, lipids, and ions. There was no natural auxotrophy reported for the organism. Hence, these biomass precursors should be produced from the central carbon pathway rather than uptake reactions from in silico media. The biomass equation comprised of 47 metabolites involved in biomass synthesis. The defects or gaps in biosynthetic pathways for these metabolites would block any biomass synthesis. The detailed set of inconsistency issues and their corrections has been provided in Table S8 (Supplementary Information). 54 reactions were added to the model with reference from the KEGG pathway database and reference model iMM904 database which finally enabled the organism to produce all the biomass precursors i.e. nucleotides, amino acids, carbohydrates, lipids and energy equivalents just from glucose, oxygen, ammonium salt and trace elements. A sensitivity analysis was conducted on the biomass equation to evaluate its robustness to fluctuations in biomass composition (Fig. 2C). This was necessary because the biomass equation was obtained from iMM904 model. In this analysis, the biomass precursor coefficients were varied by ± 50% and a FBA was run to estimate the growth rate. The specific growth rate was most sensitive to 1,3 Beta-Glucan and least sensitive to Zymosterol. The varied sensitivity of the growth rate could be attributed to the percentage contribution of the biomass precursor towards the biomass equation. The distribution of these gap filled reactions in different compartments of the metabolic model has been presented in Fig. 1C. In internal reactions, the major inconsistencies were in mannan biosynthesis and fatty acid production pathways. Mannan biosynthesis takes place in the endoplasmic reticulum facilitated by dolichol phosphate anchoring^34^. The major gaps in this pathway comprised of transport reactions between the cytoplasm and endoplasmic reticulum. It was rectified manually with reference from KEGG and MetaCyc. The carbon conversion yield for mannan on glucose was 0.0728 g/g for *L. kluyveri* (iPN730). The mannan yield on glucose for *S. cerevisiae* (iMM904) was around 0.0214 g/g. We anticipate the differences between the values could be directly due to the differences in metabolic objectives of both the organisms. *S. cerevisiae* has definite tradeoffs between biomass and ethanol production. It prefers the production of ethanol even in aerobic conditions (Crabtree Effect). Therefore, in the case of *S. cerevisiae* there is a reduced diversion of glucose into biomass at physiological uptake rates, compared to *L. kluyveri*. Similarly, the fatty acid pathway comprises of elongation of long-chain fatty acid with the addition of an even number of carbon through malonyl-CoA^35^. Due to this, any reaction blockage owing to incorrect flux direction or missing metabolites could render no flux through the whole pathway*.* We therefore, manually checked through the whole pathway to ensure that no reaction is blocked. By gap filing the fatty acid pathway manually, we enabled the model to produce basic fatty acid products i.e. phospholipids, diacylglycerol, etc. To find the blocked reactions, the corresponding reactions in the fatty acid pathway were set as the objective function and were checked for any flux through them. Iteratively all the blocked reactions were identified in the pathway and corrected using MetaCyc and KEGG as a reference. Additionally, there were significant inconsistencies in the transport of gases across the compartments. The diffusion of gases from extracellular space to a specific compartment and vice versa was important. The transport of oxygen from the extracellular space to the mitochondria through the cytosol was corrected by adding the transport reactions at the cell membrane and mitochondrial membrane. Any erroneous reaction which consumes oxygen (fatty acids desaturase reactions) were removed or given the proper reaction directionality. This was to avoid the diversion of oxygen to these reactions in the cytosol which is physiologically irrelevant. The numerical confidence on the reactions in the model was assigned based on the gene-protein reaction (GPR) and observed flux through it (Table S2, Supplementary Information). This will help the users to ascertain the confidence in predictions made using the model for metabolic flux analysis.

Although *S. cerevisiae* is phylogenetically very close to *L. kluyveri*¸ it has significantly different model statistics (Table 1). *S. cerevisiae* has a higher number of duplicate genes compared to *L. kluyveri* which has been reflected in the total number of genes in their respective metabolic models i.e. 904 vs 730. For example, *S. cerevisiae* has fatty acyl-CoA synthetases i.e. FAA1 (Gene symbol: YOR317) and FAA4 (Gene symbol: YMR246W) whereas *L. kluyveri* has just one that encodes for a fatty acyl-CoA synthetase. Similarly in the annotated genome, 527 instances showed that multiple *S. cerevisiae* genes were mapped to a single locus in *L. kluyveri.* The metabolites and the reactions are distributed in various metabolic modules in the network i.e. different functionalities of the metabolic network (Fig. 1D). Majority of the metabolites and reactions belong to the amino acid metabolism attributed to their variety compared to other biomass precursors. Nucleotide and fatty acid metabolism follow the amino acid metabolism in the number of metabolites and reactions. This is primarily due to their limited biochemical diversity. Moreover, the subcomponents of these two pathways are derived from common biosynthetic precursors opposed to amino acid metabolism.

**Table 1:** Comparative analysis of *L. kluyveri* (iPN730) with other reported genome-scale metabolic reconstructions such as *S. cerevisiae* (iMM904), *C. tropicalis* (iCT646), *Y. lipolytica* (iYL647) and *K. lactis* (iOD907).

	iPN730^This study^	iMM904^36^	iCT646^37^	iYL647^38^	iOD907^25^
Reactions	1235	1577	945	1347	1867
Metabolites	1179	1226	712	1119	1476
Genes	730	905	646	647	907
Compartments	8	8	4	8	4

The GEM for *L. kluyveri* was derived from the consensus reconstruction of different published metabolic models (“Methods”). The physiologically relevant characteristics i.e. formula, charge, and reversibility were assigned to metabolites and reactions. Incomplete biosynthetic pathways derived from the genome annotation were corrected to enable the synthesis of all essential biomass precursors. This qualified the model for further downstream applications to predict the flux distribution in the pathway in physiological conditions. The species-specific reactions like ethyl acetate metabolism and pyrimidine degradation routes were incorporated into the model. The model also mapped the genotype of the organism to a complex phenotype like flux distribution in the metabolic network which can be used to predict the phenotypic changes in the organism in response to combinatorial genetic perturbations.

### Model validation through flux balance analysis

Flux Balance Analysis (FBA) solves a linear optimization problem with the objective as the specific growth of the cell i.e. flux through the biomass equation. This assigns fluxes to active reactions in the model given the constraints. The constraints of the optimization are defined by the bounds on the flux through the reactions and the uptake rate of substrates and other media components. This is further dictated by the thermodynamic Gibbs free energy (∆G) of the metabolic reactions.

From the previously generated experimental data^18,39,40^, the accuracy of the prediction of the model was evaluated. Using the similar substrate and oxygen uptake rate as measured in the experiment, the specific growth rate and the product secretion rates were evaluated in silico by FBA. In the experiments reported previously, for a glucose uptake rate of 2.28 mmol/gdw/h and an oxygen uptake rate of 6.2 mmol/gdw/h, a specific growth rate of 0.2/h were observed^18^. This corresponded to a carbon dioxide production rate of 6.4 mmol/gdw/h and a respiratory quotient (RQ) (the ratio of the volume of carbon dioxide evolved to that of oxygen consumed) of 1.03. To this, the predicted specific growth rate and the respiratory quotient were compared to the experimentally measured values^18,41^ (Table S1, Supplementary Information). For the specific growth rate, the analysis yielded a Pearson’s correlation coefficient of 0.96 and a p-value of 0.0021 (Fig. 2A). For the respiratory quotient, the Pearson’s correlation coefficient value was 0.97 and a p-value of 0.033 (Fig. 2B). Usually, the respiratory quotient depends on the respiratory and the fermentative metabolism of the cell. The RQ values vary in aerobic and anaerobic conditions depending on the metabolic state of the cell. A high correlation between experimental and in silico measurements validated the correct behaviour of the model in the span of all possible metabolic states. The bi-modal nature of the distribution in histograms of growth rate and RQ values is due to the growth of the organism lying in the aerobic and anaerobic growth regimes (Fig. 2A,B). The growth rate in aerobic conditions is higher compared to anaerobic conditions. The studies used for validation of the simulation contains information about the growth and metabolism in different sets of conditions both in continuous and batch mode. The axes histograms (Fig. 2A,B) describe the frequency distribution of various growth rates/respiratory quotients in the experimental data (Table S1, Supplementary Information). The bimodal nature of the histograms for either cases present the differential metabolism of the organism when it lies in aerobic and anaerobic regimes.

**Figure 3:**
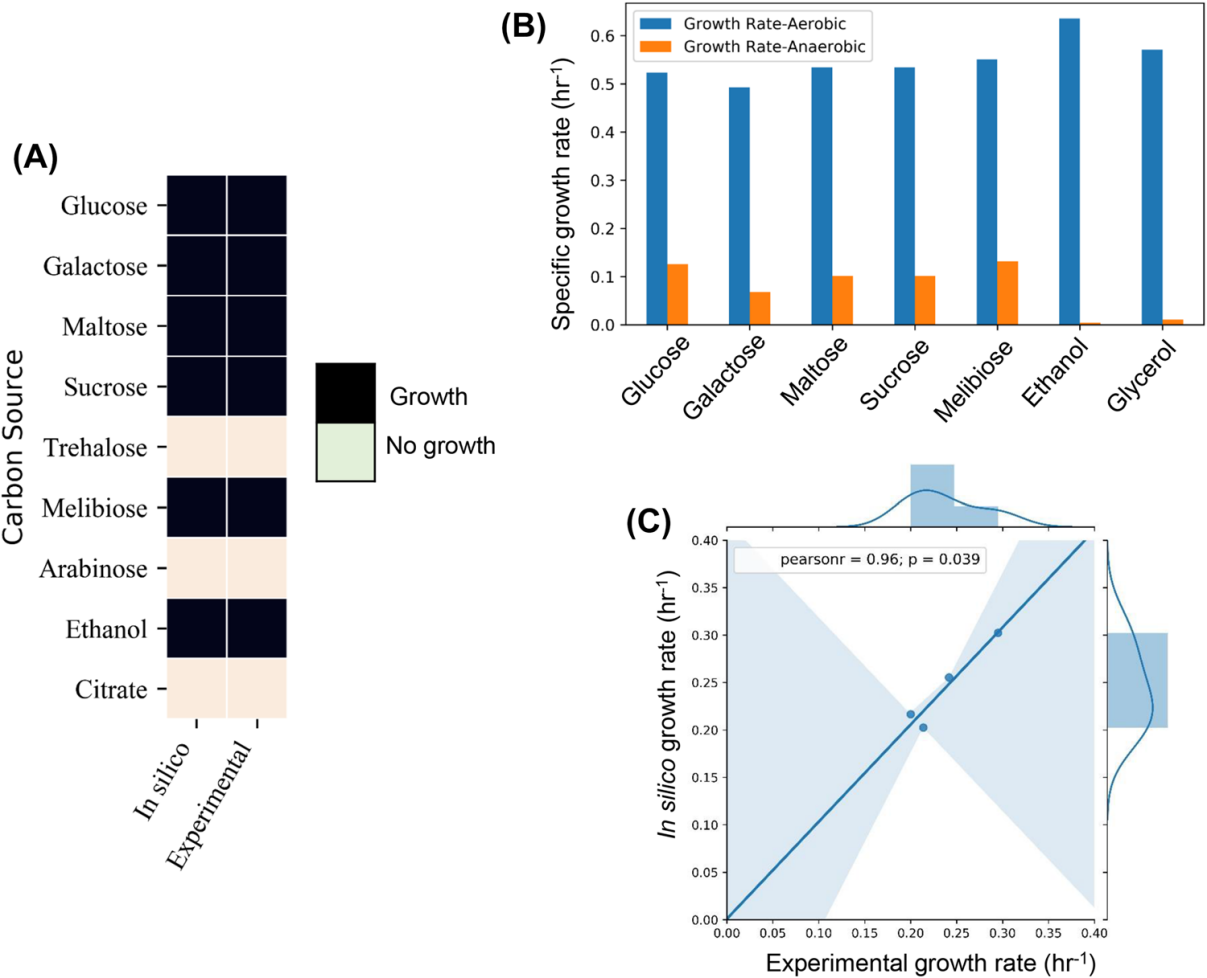
Viability and growth on various carbon sources in comparison to experimental reports. (**A**) Comparative analysis of in silico viability on multiple carbon sources and the reported data in MycoBank and other literature. (**B**) The in silico specific growth rate (h^−1^) simulated by FBA of *L. kluyveri* on different carbon sources in both aerobic and anaerobic conditions. The error bars represent the maximum and minimum specific growth rate for flux variability analysis. (**C**) The correlation between the experimental growth rate and in silico growth rate on various carbon sources.

The in silico growth and the viability of the model were evaluated in different carbon sources (Fig. 3A). The carbon sources considered were glucose, galactose, maltose, sucrose, trehalose, melibiose, arabinose, ethanol, and citrate. The in vivo ability of *L. kluyveri* to grow in multiple carbon sources was procured from MycoBank^42^. The in silico viability showed agreement with the reported in vivo viability (Fig. 3A). With an uptake rate of 1 g/gdw/h, the in silico growth rate on various carbon sources like glucose, galactose, maltose, sucrose, melibiose, ethanol and glycerol was evaluated in aerobic and anaerobic conditions (Fig. 3B). The highest in silico growth rate in aerobic conditions was for ethanol followed by glycerol and glucose. The lowest growth rate was for galactose. The growth rate in anaerobic conditions was proportional to aerobic conditions for glucose, galactose, maltose, sucrose and melibiose. In an interesting observation, the growth rate on glycerol and ethanol was higher compared to others in aerobic conditions but the lowest in anaerobic conditions. This can be explained by the redox chemistry of ethanol and glycerol utilization. Both of them are products of the central carbon metabolism formed by reduction of glycolytic intermediates i.e. dihydroxyacetone phosphate^43^ and acetaldehyde respectively. Ethanol and glycerol are non-fermentable carbon sources due to the redox imbalance caused during the anaerobic conditions. NADH redox imbalance in anaerobic glycerol metabolism impedes the growth^44^. Similarly, metabolic assimilation of ethanol in anaerobic conditions causes NADH imbalances due to the production of excess NADH which cannot be oxidized due to the absence of oxygen. We also performed batch growth experiments to validate the prediction of the growth rates on various carbon sources. The organism failed to grow on glycerol as the sole carbon source in shake flask experiment. This could be due to requirement of complete aerobic conditions in shake flask experiment. We could grow the organism on maltose, sucrose and galactose (Table S6, Supplementary Information) and measured the specific growth rate and substrate uptake rates (“Methods”). Using this, we showed a high correlation between the experimental growth rate and the predicted growth rate (Fig. 3C).

**Figure 4:**
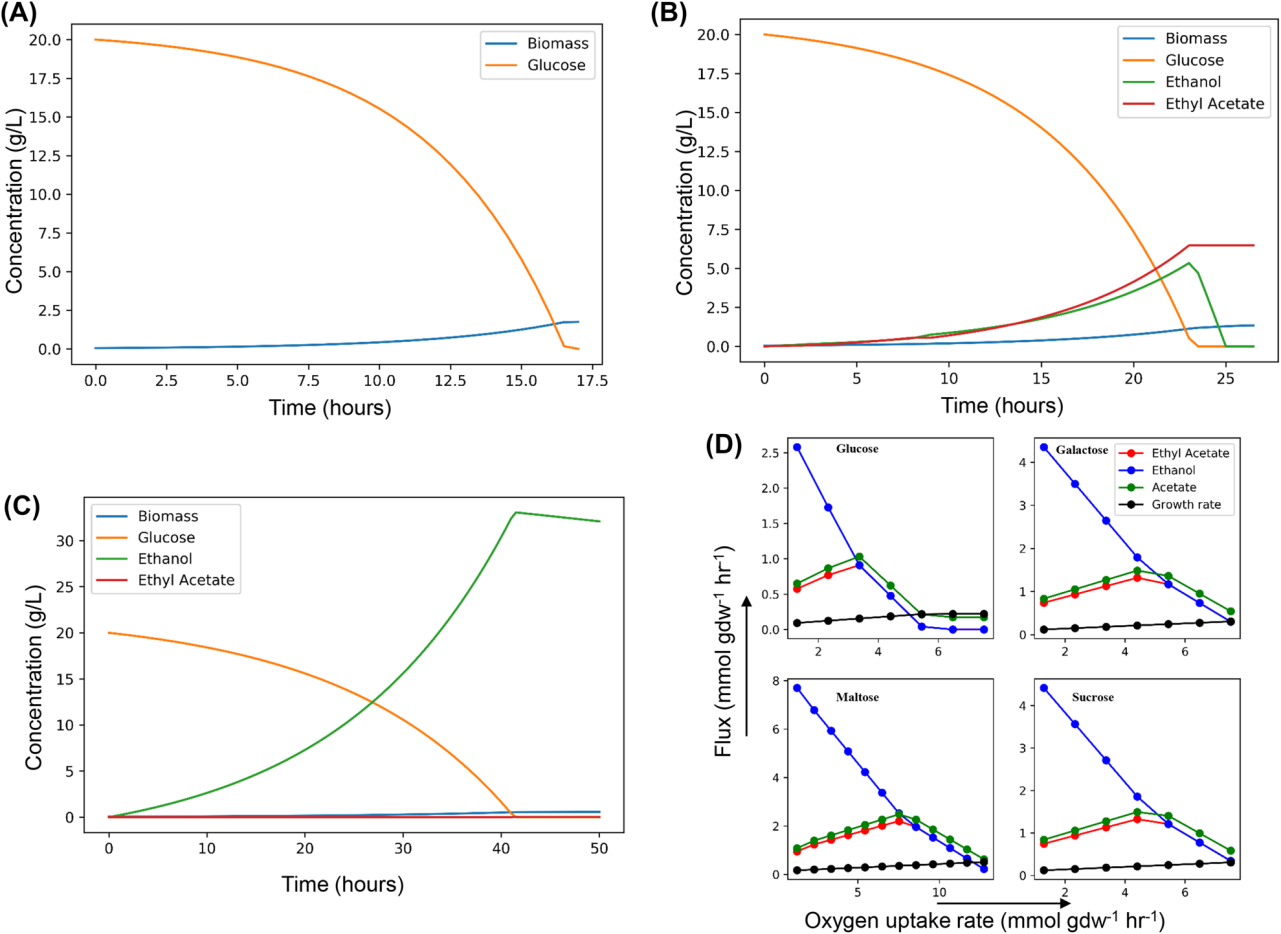
The dynamic flux balance analysis of iPN730 in oxygen limited conditions. DFBA on glucose as carbon source and oxygen uptake in (**A**) aerobic (**B**) semi-aerobic and (**C**) anaerobic regime. (**D**) The shift from respiratory metabolism (20 mmol gdw^−1^ h^−1^) to fermentative metabolism (0.25 mmol gdw^−1^ h^−1^) on glucose, maltose, sucrose and galactose shows the variation in production of acetate and ethanol which are precursor for the major overflow metabolite ethyl acetate.

The substrate consumption, growth and product formation profiles were obtained from dynamic flux balance analysis (DFBA). DFBA^29^ comprises of iterative flux balance analysis on a given amount of carbon source. A glucose uptake rate of 2.28 mmol/gdw/h was considered as reported to be a realistic uptake rate^18^. In aerobic conditions with an oxygen uptake rate of 1000 mmol/gdw/h, the model did not produce any significant metabolite apart from biomass (Fig. 4A). It agrees with the experimental results because *L. kluyveri* in aerobic conditions produces insignificant amounts of ethanol due to weak Crabtree positive nature. In semi-aerobic conditions with an oxygen uptake rate of 2 mmol/gdw/h, the model produced ethyl acetate and ethanol apart from biomass (Fig. 4B). The production of ethyl acetate as a major overflow has been reported in previous studies^2^. The ethanol concentration increased to a point until glucose was present in the medium after which it was consumed as a carbon source to fuel the growth. In anaerobic conditions with a very low oxygen uptake rate of 0.25 mmol/gdw/h, the ethanol production increased significantly while ethyl acetate production was lowered compared to semi-aerobic conditions (Fig. 4C). Similar to semi-aerobic conditions, the ethanol was used as the sole carbon source once glucose was exhausted in the medium. The reduction in the production of ethyl acetate both in complete aerobic conditions and in anaerobic conditions can be attributed to a reduction in the production of either of its precursors i.e. acetate and ethanol. Ethanol production was reduced in aerobic condition and acetate production was reduced in anaerobic conditions due to diversion of glycolytic flux towards ethanol. This is attributed to the rate of ethanol production increasing and acetate production decreasing with a decrease in oxygen uptake rate. The production kinetics of ethyl acetate and ethanol under varying carbon sources and the oxygen uptake rate was further estimated. From a discrete range of oxygen uptake rates, the model was subjected to FBA. The uptake rates for the carbon sources were constrained using the experimental values in Table S6 (Supplementary information). The ethanol and ethyl acetate production kinetics for various conditions has been given in Table S7 (Supplementary Information). The peak production rate for ethyl acetate for different carbon sources was maximum for maltose (~ 2.20 mmol/gdw/h) and the least for glucose (~ 0.91 mmol/gdw/h). The high production in the case of maltose is due to the high carbon influx into the cell (~ 26.52 mmol C/gdw/h ) compared to low carbon influxes in other carbon sources i.e. galacose: 14.16 mmol C/gdw/h, glucose: 13.68 mmol C/gdw/h, sucrose: 14.4 mmol C/gdw/h. Higher ethyl acetate production rates in the case of galactose (~ 1.32 mmol/gdw/h) compared to glucose (~ 0.91 mmol/gdw/h) might be due to the higher substrate uptake rate (Table S6, Supplementary Information) in the case of galactose. In none of the cases, ethyl acetate was produced in anaerobic condition. This is specifically due to the lack of acetate flux in anaerobic condition. For each of the carbon source, the oxygen demand for peak ethyl acetate production was determined (Fig. 4D). In the case of glucose, for peak production of ethyl acetate the oxygen demand was the lowest (~ 3.36 mmol/gdw/h) and it was higher for maltose (~ 7.52 mmol/gdw/h). This clearly depicts the correlation between high oxygen uptake rate, high carbon flux and ethyl acetate production. In comparison to the experimental data^2,18^, the DFBA predictions show a considerable agreement (Fig S1, Supplementary Information). The reported yields for ethyl acetate and ethanol were in the range 0.08–0.1 g/g glucose when the experiment was conducted in batch system with 20 g/L glucose. FBA using a range of oxygen uptake rate covering the aerobic, semi-aerobic and anaerobic regions helped us estimate the theoretical metabolite production rates (Fig S1, Supplementary Information). In the aerobic regimes (OUR ~ 2.5–3 mmol/gdw/h), the ethanol yields are around 0.055 ± 0.02 g/g glucose (experimental ~ 0.09 ± 0.01 g/g glucose) and ethyl acetate yields around 0.18 ± 0.02 g/g glucose (experimental ~ 0.12 ± 0.01 g/g glucose).

The model was able to predict the growth rate and the respiratory quotient with good accuracy in comparison to the experiments. The ability to grow and ferment on melibiose differentiates the organism from the related yeasts^3^. The variation in growth rate while growing on different carbon sources can be attributed to the energetic cost and the benefit of metabolizing them. Ethyl acetate and ethanol production phenotype observed in in silico growth kinetics showed agreement with the reported overflow metabolite production. This model can be potentially leveraged to generate hypotheses in relation to the metabolism and physiology of the organism.

### In silico single knockout analysis shows an agreement with experimental data

**Figure 5:**
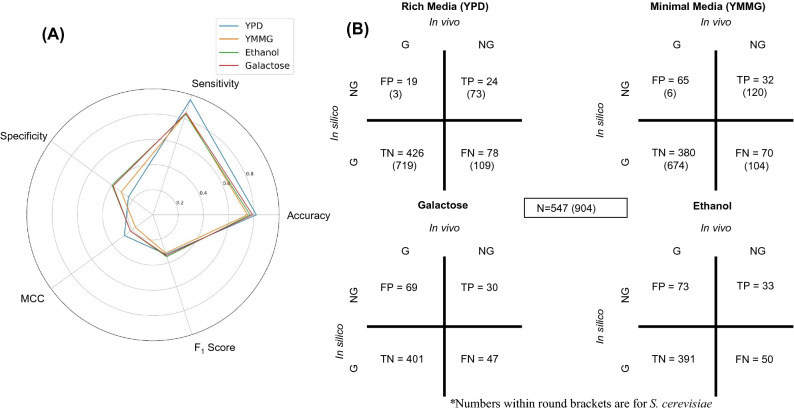
Single knockout analysis of viability in rich and minimal media. (**A**) Radar plot for comparative analysis of the model in rich media with glucose, minimal media with glucose, minimal media with ethanol and minimal media with galactose for classifying essential and non-essential genes over multiple statistical parameters i.e. Accuracy, Sensitivity, Specificity, MCC and F_1_ score. (**B**) Cross comparison between ground truth (*Saccharomyces cerevisiae* gene essentiality data) and the model predictions in the given media conditions. NG and G stands for No Growth and Growth respectively for each in silico knockout.

Genome-scale metabolic models map the genotype of an organism i.e. gene information to the metabolic flux phenotypes of the cell. Each gene encodes for protein/subunit of a protein that catalyzes a specific reaction^22^. Each of the reactions is assigned a flux solution space through FBA. This enables the metabolic model to predict the changes in flux phenotypes with respect to perturbation in genotype i.e. single knockout or combinatorial knockout analysis. To gain a high degree of confidence, it was imperative to validate such predictive power of the model in comparison to experimentally established datasets. The genotype information was mapped to the reaction information through Boolean logic. For encoding a protein complex, either all genes or only some of them are required. This is well presented by the Boolean logic using AND or OR to map the genes to the protein. The viability should remain unaffected by the deletion of genes that do not have obligatory functions. Two large scale gene knockout studies in *S. cerevisiae* were used to evaluate the predictive power of the model. Comparative gene knockout analysis of the model with reference to the published datasets^28,45^ for homologous genes in *S. cerevisiae* was performed. Statistical analysis was performed in terms of sensitivity, specificity, F_1_ score and Mathews Correlation Coefficient (MCC) (Fig. 5A). FBA and MOMA^46^ (Minimization of Metabolic Adjustment) were performed to assess the viability of the model based on single-gene knockouts. Due to the similar performance of both the approaches i.e. FBA and MOMA, only FBA was considered for any further discussion.

When cells grow in minimal medium where only carbon source, ammonium salts, phosphate and trace elements are present, it requires all the enzymes necessary for synthesizing amino acids, nucleotides, lipids and carbohydrates. In rich media, the cells are supplied with all the growth precursors in a platter so the cells relax the biosynthesis of those precursors^47^. In the genome-wide single-gene knockout analysis, the metabolic model supplemented with all growth precursors has significantly fewer essential genes i.e. true positives compared to growth on minimal media (Fig. 5B). The total number of true essential genes were calculated with reference to the corresponding single knockout dataset of *S. cerevisiae* (Table S3, Supplementary Information)^28,45^. Only 74.93% (547) genes had a homolog in *S. cerevisiae*. The model had a sensitivity of 0.58 for rich media and 0.32 for the minimal media. It had almost equal specificity scores of 0.84 in rich and minimal media. For evaluating the performance of the model in classifying essential and non-essential genes, F_1_ score and MCC were used. F_1_ score for predictions was 0.324 in rich media, 0.317 in minimal media with glucose. MCC for predictions was 0.28 in rich media, 0.162 in minimal media with glucose. The radar plot (Fig. 5A) compared the statistical figures of merit when the single knockout analysis was performed in minimal with glucose or ethanol or glucose and rich media. *S. cerevisiae* single-gene knockout database was used due to the unavailability of extensive knockout analysis for *L. kluyveri*. A considerable F_1_ score and MCC being on the higher side for the model validated the confidence in the predictions. The gene essentiality analysis was conducted for YPD, synthetic yeast media with glucose, synthetic yeast media with galactose and synthetic yeast media with ethanol. All the analysis was conducted in complete aerobic conditions where oxygen uptake rate was set in the non-limiting region. The accuracy, sensitivity, specificity, F_1_ score and MCC in the case of galactose and ethanol were comparable to the growth on YPD and glucose minimal media. The gene essentiality statistics (Fig. 5B and Table S3, Supplementary Information) are different for carbon sources. This is due to utilization of different metabolic modules during the growth on different carbon sources. The number of false positives in the case of galactose and ethanol were comparable to that of glucose minimal media. This has been attributed to the duplicate genes present in *S. cerevisiae*. Moreover, growth of YPD supplies the cells with all the essential macromolecule precursors hence suppressing the effect of presence of duplicate genes. Due to this, we get significantly less number of false positives in the case of YPD i.e. 19 compared to other growth conditions i.e. glucose minimal (65), galactose minimal (69) and ethanol minimal (73)*.* The gene essentiality prediction statistics for *S. cerevisiae* (iMM904)^36^ for YPD and glucose minimal media has been added for comparison to *L. kluyveri* (Fig. 5B). The total frequency of genes in the case of iMM904 was 904 whereas that of *L. kluyveri* was 547. Both in the case of *L. kluyveri* and *S. cerevisiae*, the fraction of essential genes truly predicted was < 10%. There were significantly high number of false positives in *L. kluyveri* compared to *S. cerevisiae* even after we represent it as a fraction of total genes. This could be directly attributed to the duplicate genes in *S. cerevisiae* compared to *L. kluyveri*. This comes up as a limitation of using in vivo knockout data for in silico comparison with model simulation between two species having ortholog duplicates.

### Integration of reported metabolic routes in the model iPN730

**Figure 6:**
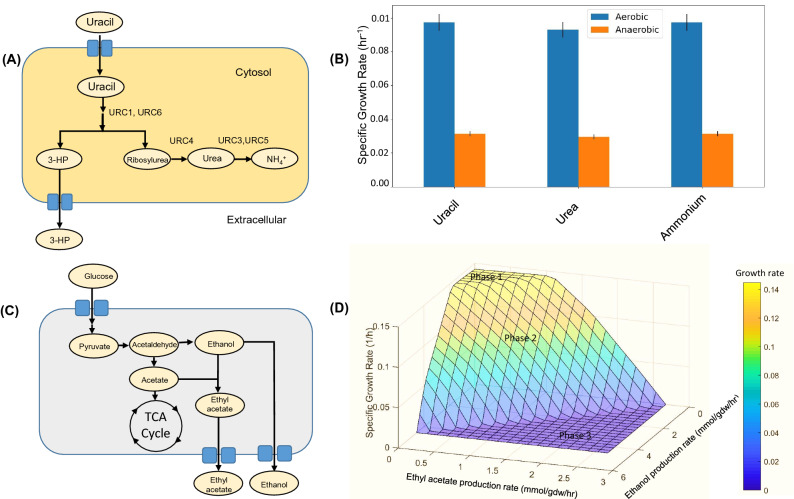
URC pathway for pyrimidine degradation in *Lachancea kluyveri.* (**A**) URC pathway in *L. kluyveri* reported in literature. This enables the organism to assimilate Uracil and other pathway intermediates as its sole nitrogen source. (**B**) The fitness in terms of specific growth rate for iPN730 simulated on uracil only or urea only or ammonium only as the sole nitrogen source. The bars represent 5% variation in the specific growth rate. (**C**) Ethyl acetate pathway inferred from iPN730. The acetate is derived from the central carbon metabolism whereas the ethanol is derived from the fermentative metabolism. AAT is the principal enzyme that fuses both together to form ethyl acetate. (**D**) Phenotypic Phase Plane Analysis of ethyl acetate and ethanol production in *L. kluyveri* with respect to its growth rate. The analysis was conducted in semi-aerobic condition i.e. OUR of 3 mmol/gdw/h. Phase 1 denotes the optimal growth region. Phase 2 denotes the region where the ethyl acetate and ethanol production penalizes growth rate. Phase 3 denotes the infeasible regions with negligible growth rate.

In the previous published literature, important metabolic phenotypes for *L. kluyveri* have been reported. Of the four possible modes of pyrimidine degradation known, the uracil (pyrimidine) catabolism (URC) pathway has been uniquely characterized in *L. kluyveri*^1,4,48^*.* Locus URC1-6 and URC8 have been experimentally characterized to be an essential component of the URC pathway for uracil degradation. This enables *L. kluyveri* to grow on uracil and the intermediates of the degradation pathway as the sole nitrogen source. This ability is supposedly lost in *S. cerevisiae* after the whole genome duplication event^4^. In the URC pathway, the gene products of URC1 and URC6 degrade uracil to 3-hydroxypropionate and ribosylurea through multiple steps that have not been characterized. The gene product of URC4 degrades ribosylurea to urea. The urea is further broken down by the action of gene products of URC3 and URC5 to ammonia and carbon dioxide. The ammonia/ammonium then replenishes the nitrogen pool for amino acid and nucleotide biosynthesis. This enables the organism to grow on uracil and urea as the sole nitrogen source. The whole pathway (Fig. 6A) was incorporated into the metabolic model iPN730. The permeases/transporters for the intermediates of the pathway were incorporated into the model to enable their uptake. The ability of the model to show in silico growth on uracil and urea as a nitrogen source was evaluated. The model was able to incorporate nitrogen from uracil and urea into its metabolism. The uptake rate for uracil and urea per unit uptake flux of glucose in complete aerobic conditions was 1.009 mmol/gdw/h and 0.261 mmol/gdw/h respectively. With ammonium as the sole nitrogen source, the uptake rate per unit uptake flux of glucose was 0.526 mmol/gdw/h (Fig. 6B). We also found a high agreement between the in silico growth rate (0.097/h) and the experimental growth rate (0.0962/h) (Table S6, Supplementary Information).

The ethyl acetate biosynthesis pathway was reconstructed in iPN730 which was reported earlier*.* Its production was highest in the semi-aerobic condition when ethanol production and acetate production were optimal. Ethyl acetate pathway comprises of a transporter (hypothetical) and an enzyme alcohol acetyl transferase (AAT) (Fig. 6C). AAT catalyzes the fusion of ethanol and acetate into ethyl acetate. It is encoded by PCCGCIGL_06287 gene obtained from genome annotation. The ethyl acetate is transported to the extracellular space using a hypothetical ethyl acetate transporter. A transport reaction for ethyl acetate was incorporated into the metabolic model that enabled its secretion. The transition from a growth regime to the bioproduction regime was captured by the Phenotypic Phase Plane (PPP) analysis (Fig. 6D). The PPP comprised of three phases in the case of ethyl acetate and ethanol production in *L. kluyveri*. Phase 1 was the optimal growth regime where there was physiological production rates for both the compounds. In this region, the production rates of ethyl acetate and ethanol was coupled with the biomass production. Phase 2 comprised on the region where the detrimental effects of ethyl acetate and ethanol overproduction on the growth rate is evident. Phase 3 comprises of the region where the energetic costs of their production reaches the cellular limits. PPP analysis helped in understanding the penalties on the growth rates due to the overproduction of the target compounds ethyl acetate and ethanol.

Pyrimidine degradation pathway in *L. kluyveri* has been discussed in relation to anti-cancer drug design as understanding pyrimidine homeostasis directly affects the DNA replication machinery^4^. Although the pyrimidine catabolism pathway has been sufficiently characterized, iPN730 will give an opportunity to understand the genotype to flux phenotype relations and its interaction with other pathways at a systems level.

### Context specific versions of iPN730 highlight pathways associated with uracil degradation

The context specific model of *L. kluyveri* growing on either ammonium or uracil as the sole nitrogen source was prepared using GIMME algorithm^49^. The gene expression data (GSE48135)^48^ was used here for building the context specific model of *L. kluyveri* by eliminating the inactive reactions. The expression was measured in wild type *L. kluyveri* NRRL 12,651 in minimal media with either ammonium or uracil as the sole nitrogen source. There would be several reactions activated or deactivated between both the conditions which would alter the metabolic flux through pathways. The ability of *L. kluyveri* to use uracil as the sole nitrogen source comes from its unique pyrimidine catabolism pathway^4^. We earlier integrated the pathway in *L. kluyveri* and validated the flux balance analysis prediction of growth rate (Table S6, Supplementary Information). Integration of the gene expression data using GIMME algorithm at a gene expression threshold of 25th percentile reduced the number of active reactions in both the models. In the context specific model for growth on uracil (iPN730_U), the number of reactions were 1089 and the number of metabolites were 1106. In the context specific model for growth on ammonium salt (iPN730_A), the number of reactions were 1099 and the number of metabolites were 1119. There were 78 unique reactions in the case of iPN730_U and 88 unique reactions in the case of iPN730_A. The remaining reactions were common in both.

**Figure 7:**
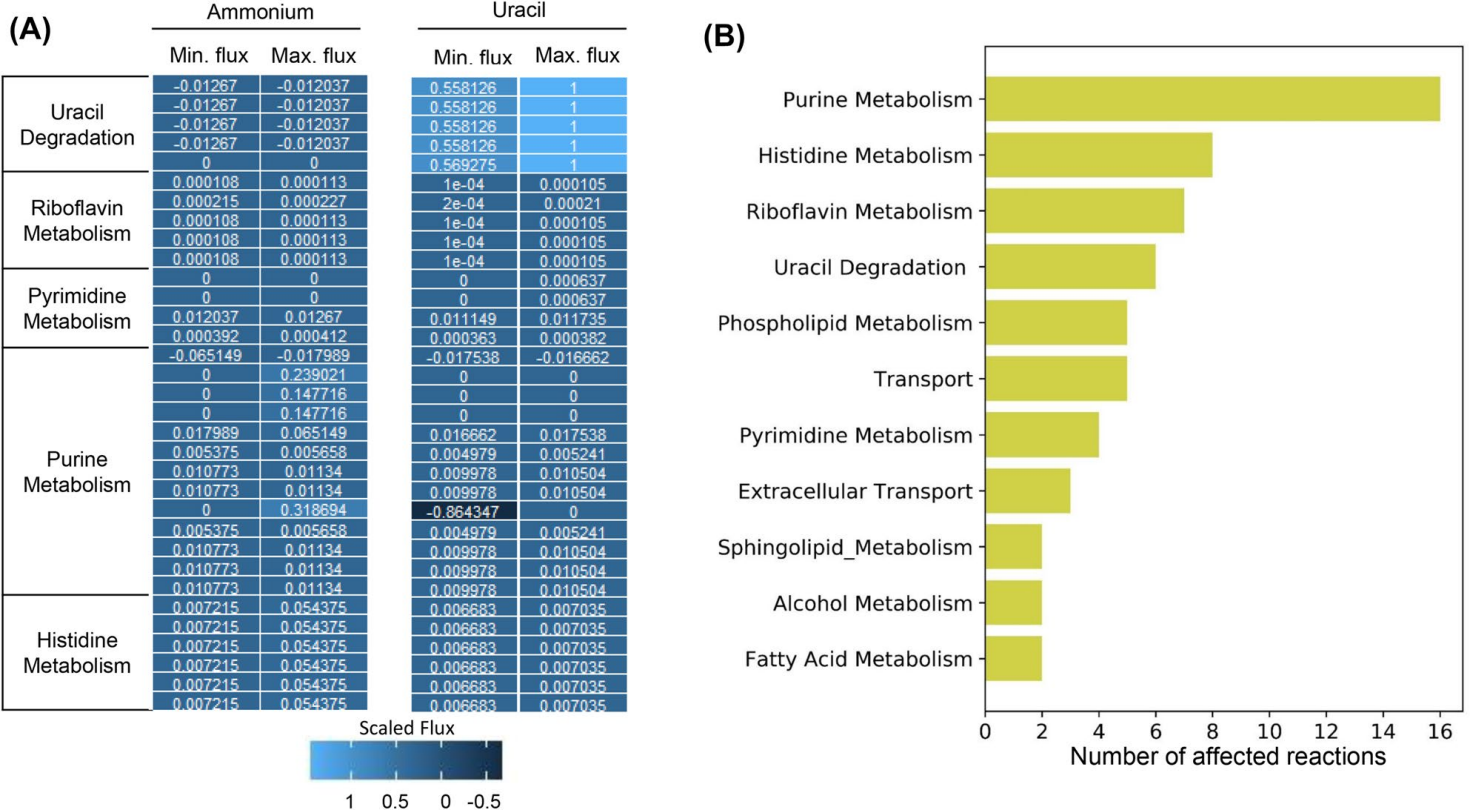
Context specific metabolic model of iPN730 for studying pyrimidine degradation. (**A**) Loopless flux variability analysis of the context specific model of *L. kluyveri* growing on ammonium or uracil as the sole nitrogen source. The pathways associated with the flux are given in the figure. (**B**) Pathways impacted by the growth on uracil compared to growth on ammonium as the sole nitrogen source. The pathways are ranked based on the number of constituent reactions that are affected. FVA along with Jaccard index was used to infer the affected reactions.

Flux Variability Analysis (FVA) allows to explore the bounds of the flux space under constrained conditions^50^. It gives a minimum and maximum flux value for each reaction in a given condition. While flux calculated by FBA might not be unique for an objective value, FVA gives a confidence interval for the flux values. This allows us to compare the flux between two different conditions or models. FVA usually results in formation of futile cycles inside the model which gives erroneous flux predictions. Using loop-less mode of FVA, eliminated the formation of such futile cycles in both of the models. The FVA ranges were then compared between both the models to predict the reactions having differential flux when grown on uracil with respect to growth on ammonium (Fig. 7A). The impacted reactions as identified by Jaccard index were clustered based on their parent pathways. We considered all those reactions having a Jaccard index of zero between both the models as having differential flux. Around 74% of the reactions having differential flux belonged to five pathways (Fig. 7B) i.e. (1) Purine metabolism (2) Histidine Metabolism (3) Riboflavin Metabolism (4) Pyrimidine Metabolism (5) Uracil Degradation. Rest of the affected reactions belong to (1) Phospholipid metabolism (2) Sphingolipid metabolism (3) alcohol metabolism pathway.

Uracil degradation not only replenishes the nitrogen pool of the cell, it also feeds into the pyrimidine pathway allowing for its utilization in nucleotide salvage pathway which generates the nucleotide precursors^4^. Purine metabolism pathway has multiple reactions affected when the cells are growing on uracil with respect to growth on ammonium. This is well explained by the high level of interaction between the pyrimidine pathway to which uracil belongs and the purine biosynthesis pathway. Both the pathways share the same precursor i.e. Inosine Monophosphate (IMP) for de novo biosynthesis of the purines and pyrimidines. It is expected that any flux difference in uracil metabolism is going to interfere with the flux values in the interacting pathways. Similarly, Histidine metabolism is closely connected with nucleotide biosynthesis pathway^51^. Both the pathways share the same route beginning from Phosphoribosyl Pyrophosphate (PRPP) to Phosphoribulosylformimino-AICAR phosphate which leads to the formation of IMP in the case of nucleotide biosynthesis pathway. The flux through the Histidine biosynthesis pathway is therefore altered when growing on uracil as the sole nitrogen source. GTP serves as the immediate precursor to riboflavin biosynthesis pathway^52^. Therefore, it is evident that an altered purine biosynthesis pathway to which GTP belongs, will impact the biosynthesis of riboflavin. Riboflavin is also associated with folate biosynthesis pathway which interacts with nucleotide biosynthesis pathway by feeding one carbon pool like methyl and formyl groups. It is interesting to note that phospholipid pathway is also affected to a considerable extent due to growth on uracil compared to ammonium. A change in flux through Acetyl CoA Carboxylase (ACC1) which is the first committed step of fatty acid biosynthesis is also affected. Nucleotide products not only serve as a precursor pool to the DNA and RNA biosynthesis, they are also involved in energy metabolism and phosphate transfer to other biosynthetic intermediates. Phospholipids are one of such intermediates which has a high demand for phosphate groups which are supplied by the nucleotides. Uracil degradation pathway is activated when the model is simulated on uracil as the sole nitrogen source (Fig. 6A).

Pyrimidine degradation pathway is uniquely present in *L. kluyveri* compared to all its neighboring yeasts. Its importance has been highlighted in the context of anti-cancer drugs development which target nucleic acid metabolism like 5-fluorouracil. This drug degrades due to the pyrimidine degradation pathway in human cells. It would therefore be imperative to study remedies for this. The current analysis highlighting the interactions between pyrimidine degradation and other associated pathways would serve as an essential piece of information.

## Discussion

The first genome-scale metabolic model for *L. kluyveri*, iPN730, reflects important phenotypes in this organism that have been experimentally characterized earlier. Model integration statistics reveal *L. kluyveri* to be closer to *S. cerevisiae* and *C. tropicalis.* There are 527 instances of duplicate genes in *S. cerevisiae* compared to *L. kluyveri* owing to the whole genome duplication event that took place in the evolutionary time course. The knowledge of the pyrimidine degradation pathway has been utilized for understanding the drug metabolism in the case of cancer cells particularly in relation to DNA replication machinery. The metabolic flux analysis of such pathways can be quintessential in understanding the dynamics of homologous pathways in higher eukaryotes. Due to the weak Crabtree positive metabolism, *L. kluyveri* is able to produce more biomass than *S. cerevisiae* that makes it a lucrative organism for the production of proteins and other growth associated commercially important macromolecules. The in silico viability and the growth rate in multiple carbon sources also show good agreement with the experimental data. The single-gene knockout analysis also validated the predictive power of the model iPN730 for alterations in flux phenotypes in varying genetic backgrounds. To validate the choice of objective function, we calculated the correlation between the predictions of the model and the experimental data under identical uptake conditions. We obtained a correlation of 0.96 for prediction of glucose uptake rate and 0.97 for respiratory quotient. Moreover, we also ran in silico single knockout screens on glucose, galactose and ethanol. Although we used *S. cerevisiae* datasets for evaluating the model’s prediction, we obtained an accuracy > 0.75 for all our test cases.

Due to the presence of duplicate genes in *S. cerevisiae* compared to *L. kluyveri*, the single knockout analysis might suffer some gaps. The number of false positives in the case of all the media conditions is high compared to corresponding values for *S. cerevisiae*. While a gene having a single copy might be lethal in *L. kluyveri*, the same gene in *S. cerevisiae* being duplicated buffers the lethal effect of the deletion on the fitness. This effect might be significantly suppressed when the organism is growing on rich media i.e. YPD. The organism is served with all the precursors for biomass in a platter which slacks the essentiality of many genes. Thus we observe significantly less number of false positives in *L. kluyveri* growing on YPD.

Currently, ethyl acetate market stand at $3.7 billion^53^ and its demand has a CAGR (Compounded Annual Growth Rate) of ~ 5%. It is an important industrial solvent and an essential component of paints. Production of ethyl acetate from microbial cell factories is an emerging avenue. Very few yeasts including *L. kluyveri* and *K. marxianus* produce ethyl acetate. Metabolic engineering efforts have enabled *Escherichia coli* and *S. cerevisiae* to produce ethyl acetate with considerable yields but there is significant room for improvement. *L. kluyveri* naturally produces 0.12 g/g glucose of ethyl acetate in aerobic conditions and targeted metabolic engineering can further improve the yields. In this regard, iPN730 will potentially play an important role of target metabolic engineering leveraging constraint based analysis tools. The production simulations of ethyl acetate and ethanol on different carbon sources under varying uptake rates helped in understanding the high production rates in the case of maltose in semi-aerobic conditions due to high carbon influx. Moreover, phenotypic phase plane analysis helped in understanding the cost of ethyl acetate and ethanol production incurred to the growth rate. This will potentially be an important information for strain optimization for metabolic engineering. The phase 1 region represents the growth optima regime where ethyl acetate and ethanol production rates are coupled with biomass formation. Later in Phase 2, we see the detrimental effects of overproduction on the growth rates. Algorithms like OptKnock and OptForce would help us in designing strains that couple the bioproduction with growth rates even at high bioproduction rates.

Pyrimidine degradation pathway is uniquely present in *L. kluyveri* amongst its all neighboring *Saccharomyces yeasts*^1,4^. *S. cerevisiae* seems to have lost this ability during the whole genome duplication event. 5-fluorouracil is used for treatment of colorectal, breast, as well as head and neck cancer. It still remains one of the highly prescribed drugs for anti-cancer chemotherapy. More than 80% of the dose is degraded in our body due to the pyrimidine catabolism pathway^4^. It is therefore imperative to study the pyrimidine catabolism pathway and its interactions with other pathways. The results derived from flux variability analysis of context specific genome-scale metabolic models highlighted the effects of uracil uptake on multiple pathways. Some of the pathways are closely associated with uracil metabolism whereas others do not have direct connections i.e. phospholipid and alcohol metabolism. The context specific versions of iPN730 could be used to probe pathways that have epistatic interaction with uracil degradation. This could potentially open a whole new window for therapeutics supplementing 5-flurouracil and other nucleotide analogue drugs.

The pathways for phenyl acetate, 2-methylbutanol and isobutanol were also present in the reconstructed metabolic model which are important industrial chemicals^54^. Their yields can be estimated in silico per unit mole of glucose consumed for metabolic engineering purposes. We anticipate the use of this model for deriving predictions on the flux distribution in the metabolic network in varying growth conditions. This model will add up to the non-conventional yeast genome-scale metabolic reconstructions that will enable hypotheses generation for the exploration of diverse phenotypes of fungi. This will potentially lead to the discovery of new aspects of metabolism in lower eukaryotes and the design of engineering strategies to overproduce fine chemicals of industrial relevance from *L. kluyveri.*

## Methods

### Genome-scale reconstruction of *L. kluyveri*

KBase (US Department of Energy Knowledge Database)^55^ workspace was used for annotation and generation of draft metabolic model iPN730. For the genome annotation using YGA pipeline, we have applied BDBH algorithm (Bidirectional Base Hit) with an E-value cutoff of 10^–10^ and *S. cerevisiae* (S288C) as the base template model. The contigs were uploaded in a multi-FASTA format into the server. The homology table generated from the BDBH algorithm for proteome comparison can be used to predict possible duplication, inversion, deletion, insertion or synteny loci between the organisms (Table S4, Supplementary Information). Build Fungal Model was used to reconstruct the genome-scale metabolic model using 13 previously published fungal metabolic models as templates. The template metabolic models were *S. cerevisiae*-iMM904^36^, *Aspergillus oryzae*-iWV1314^56^, *Mucor circinelloides*-iWV1213^57^, *Y. lipolytica*-iNL895^27^, *Scheffersomyces stipitis*-iSS884^58^, *Penicillium rubensi-*iAL1006^59^, *Eremothecium gosypii-*iRL766^60^, *Kluyveromyces lactis*-iOD907^25^, *Komagataella phaffii-*iLC915^61^, *Neurospora crassa-*iJDZ836^62^, *C. tropicalis-*iCT646^37^, *C. glabrata-*iNX804^57^, *A. terreus-*iJL1454^63^. Among them, *S. cerevisiae-*iMM904 was chosen as the template for the reconstruction. COBRApy^32^ was used in Spyder, Anaconda (Python 3.6) for reading and manipulating the draft model in Systems Biology Markup Language (SBML) format. COBRA Toolbox v3.0 was used in MATLAB R2017b (Mathworks, Natick, MA, USA) for identifying the inconsistencies in the model. Gurobi version 8.0 (Academic license version) and GLPK solver were used for linear programing for FBA in both MATLAB and Python. The metabolite formula and charge were added using PubChem, CHEBI (Chemical Entities of Biological Interest) and BiGG database. The reaction bounds by default were reversible. The reaction bounds were corrected using iMM904 as a reference and similar reactions were mapped. Remaining reactions were manually corrected with reference from MetaCyc, KEGG and previously published reaction reversibility database^33^. KEGG pathway database was used as a reference to find gaps in the biomass precursor synthesis pathways. Inferred reactions were added wherever necessary to enable biosynthesis of all precursors^64^. Due to insufficient information on the biomass composition of *L. kluyveri,* the biomass equation for *S. cerevisiae* (iMM904)^24^ was used for all purposes. A confidence score was assigned to each reaction considered in the model. The confidence score was assigned based on the existence of a GPR for the reaction and flux through it. A high confidence score of ‘4’ was assigned to the reactions which had a GPR assignment along with significant flux through the network calculated by flux balance analysis. The confidence score of ‘4’ was also assigned for exchange reactions necessary for growth on defined media. A score of ‘3’ was assigned for reactions having significant flux through them but no GPR assigned for them. A score of ‘2’ was assigned for reactions having GPR assignment but no flux through them for growth in defined medium. A low confidence score of ‘1’ was assigned for reactions having no GPR assignment or flux through them.

### Model validation through flux balance analysis

FBA was performed using Gurobi solver in Python 3.6 (Spyder, Anaconda). In silico media was assigned to the model for FBA by assigning experimentally reported uptake rates to the exchange reactions. The glucose and oxygen uptake rates were assigned as per experimental data. The bounds for ammonium ion and trace metals were set to very high values i.e. 1000 mmol/gdw/h to remove any limitation. In silico growth rate and respiratory quotient were compared to experimental data based on the Pearson correlation coefficient. We used the ‘jointplot’ function in the seaborn visualization and analysis library in python 3.7 for Fig. 3. The Pearson’s correlation coefficient was calculated and the p-value corresponding to the hypothesis “The experimental and in silico values are positively correlated” is given. The respiratory quotient (RQ) was calculated as:

$$ RQ = \frac{Carbon dioxide \,\,production\,\, flux}{{Oxygen\,\, uptake\,\, flux}} $$

The information regarding the growth on various carbon sources was procured from Westerdijk Fungal Biodiversity Institute’s MycoBank^42^ database. The data regarding the growth of the strain on the mentioned carbon sources can be accessed from the BioloMICS database of MycoBank using *“Lachancea kluyveri*” as the identifier. The *in silico* viability was qualitatively compared with the reported experimental growth on glucose, galactose, maltose, sucrose, trehalose, melibiose, arabinose, ethanol and citrate. The *in silico* growth rate was simulated for glucose, galactose, maltose, sucrose, melibiose, ethanol and glycerol in anaerobic and aerobic conditions. For aerobic condition, an oxygen uptake rate of 1000 mmol/gdw/h was considered and for anaerobic condition, an uptake rate of 0.25 mmol/gdw/h was considered.

DFBA was implemented for the model iPN730 in COBRA Toolbox v3.0 (MATLAB, USA). The glucose uptake rate was kept fixed at 2.28 mmol/gdw/h as measured in previous studies^18^. The uptake rate of oxygen was varied from 1000 mmol/gdw/h i.e. aerobic condition (Glucose limited regime) to 0.25 mmol/gdw/h i.e. anaerobic condition (Oxygen limited regime). An uptake rate just below the optimal uptake rate in the aerobic condition of 3 mmol/gdw/h was considered as the semi-aerobic condition. The parameters considered for the dynamic flux balance (Table 2) were chosen based on experimental conditions in which batch fermentation experiments were performed^2^.

**Table 2:** Parameters considered for dynamic flux balance analysis using COBRA Toolbox v3.0 in MATLAB 2017(b).

DFBA parameters	Parameter values
Initial concentration of glucose	20 g/L
Initial biomass concentration	0.05 g/L
Fermentation time	50 h
DFBA sampling interval	0.5 h

The DFBA algorithm applies FBA at regular intervals i.e. DFBA sampling intervals on the model and assumes that the substrate uptake rate remains constant throughout the fermentation^29^. The *in silico* media composition was recalculated based on the uptake and growth rate after every sampling interval. The following equations define the growth kinetics, substrate uptake and product formation in an *in silico* batch fermentation:

$$ X\left( {t + \Delta t} \right) = X\left( t \right) \cdot e^{\mu \Delta t} $$

$$ S\left( {t + \Delta t} \right) = S\left( t \right) - v_{EX\_S} \cdot X\left( t \right) \cdot \left( {1 - e^{\mu \Delta t} } \right)/\mu $$

$$ P\left( {t + \Delta t} \right) = P\left( t \right) - v_{EX\_P} \cdot X\left( t \right) \cdot \left( {1 - e^{\mu \Delta t} } \right)/\mu $$

$$X\left( t \right),$$$$S\left( t \right) and P\left( t \right)$$ denotes the concentration of biomass, substrate and the product respectively over the course of in silico batch fermentation. $$\mu$$ is the specific growth rate in h^−1^. *v*_*EX_S*_ and *v*_*EX_P*_ are the uptake rate and the production rate for substrate and products respectively. The products considered for DFBA were ethanol and ethyl acetate. To explain the production of ethyl acetate, the fluxes associated with the precursor-compounds ethanol and acetate were analyzed in oxygen-limited conditions. The alterations in the production fluxes for ethanol i.e. R_ALCD2ir_c0 (Alcohol Dehydrogenase 1.1.1.1) and acetate i.e. rxn00507_c0 (Aldehyde Dehydrogenase, EC 1.2.1.3) with the change in the uptake rate of the oxygen was evaluated through FBA. For example, model.reactions. R_ALCD2ir_c0.flux was used to access the flux through the production reaction of ethanol in COBRApy.

### Growth experiments on various carbon sources

*L. kluyveri* NRRL 12,651 strain was used in all the experiments. The yeast was grown in Yeast Extract-Peptone-Glucose (YPD) media (10 g/L yeast extract, 20 g/L peptone, 20 g/L glucose) overnight at 30 °C shaking at 240 RPM. The cells were washed thrice in Yeast Nitrogen Base buffer (0.67%) to remove any remaining media with centrifugation at 3000 RPM for 3 min. The growth experiments were conducted in shake flask. The media containing different carbon sources comprised of Yeast Nitrogen Base with Ammonium sulphate and without amino acids (Difco, BD); 6.7 g/L, 20 g/L of respective carbon source. The carbon sources used in the experiments were Maltose (Merck), Sucrose (Merck), and Galactose (SRL). The cells were diluted to 0.2 OD in the growth media. The optical density was measured at 600 nm using Nanodrop ONE (Thermo Scientific, USA). The substrate and extracellular product concentrations were measured in High Performance Liquid Chromatography (Agilent Technologies, HPLC 1260 Infinity) equipped with an Aminex HPX-87H ion exchange column (Biorad, USA) where 5 mM H_2_SO_4_ was used as mobile phase and flowrate was 0.6 ml/minute. Briefly, 20 uL syringe filter purified sample was injected in the column. The specific growth rate ($\mu$) was calculated as,

$$ \mu = \frac{1}{{t_{final} - t_{initial} }}\log \frac{{OD_{600} \left( {t = t_{initial} } \right)}}{{OD_{600} \left( {t = t_{final} } \right)}} $$

For growth on uracil as the sole nitrogen source, we used a concentration of 0.1% (w/v)^48^. The media contained 1.7 g/L Yeast Nitrogen Base without Ammonium Sulphate and without amino acids. For all time-course experiments, the growth was measured at a time difference of 2 h. The cells were diluted to OD in the dynamic ranges of the instrument and standard curve.

### Single knockout landscape and statistical analysis

Genome-scale metabolic models link the metabolic phenotypes to the genotype of the organism through GPR association. This enables the prediction of the alterations in the metabolic phenotype of the organism in response to genetic perturbations. To gain confidence in the accuracy of the model, it is imperative to validate the predictions with reference to experimental data. Unfortunately, such a genome-scale single gene deletion dataset was not available for *L. kluyveri.* Hence, for this purpose, the *S. cerevisiae* gene essentiality dataset was considered due to the evolutionary closeness between them and high confidence in the dataset^28,45^. The gene essentiality analysis was conducted for YPD, synthetic yeast media with glucose, synthetic yeast media with galactose and synthetic yeast media with ethanol. All the analysis was conducted in complete aerobic conditions where oxygen uptake rate was set in the non-limiting regime. Gene essentiality information for *S. cerevisiae* genes was transferred to homologous genes in *L. kluyveri. *In silico single-gene knockout was performed on iPN730 by constraining the flux to zero for the reactions mapped from the genes by the GPR association information. For this, cobra.flux_analysis.single_gene_deletion() function was used in COBRApy^32^ in Python 3.6 (Anaconda, USA) and Gurobi solver was used for the optimization. The viability of the cell in terms of the growth rate was estimated by FBA for each in silico gene deletion in the model. The in silico essentiality of the genes were compared with experimental data mapped from the gene deletion studies on *S. cerevisiae* and the statistics of the predictions were calculated as follows:

$$ Specificity = \frac{TN}{{FP + TN}} $$

$$ Sensitivity = \frac{TP}{{TP + FN}} $$

$$ MCC = \frac{{\left( {TP*TN} \right) - \left( {FP*FN} \right)}}{{\left( {TP + FP} \right)*\left( {TP + FN} \right)*\left( {TN + FP} \right)*\left( {TN + FN} \right)}} $$

$$ F_{1} Score = \frac{2*Precision*Recall}{{Precision + Recall}} $$

where TP: True Positives; FP: False Positives; TN: True Negative; FN: False Negative. Accuracy is the total true positives and true negatives that are captured by the model in comparison to the experimental data (ground truth). Sensitivity is the fraction of real positives while specificity is the fraction of real negatives that is captured by the model. MCC score and F_1_ score, both, help us in understanding the quality of the model for binary classification i.e. into essential and non-essential genes. In this case, we are using these statistical tools for evaluating the quality of our model to classify essential and non-essential genes based on their effect on its fitness. F_1_ score ranges from 0 to 1. A value of 1 is attained when there is perfect prediction. MCC score ranges from − 1 to 1, where − 1 denotes absolute disagreement between ground truth and the prediction while 1 denotes perfect prediction. The single-gene deletion analysis was performed in different in silico media. Aerobic conditions in which original experiments for *S. cerevisiae* were performed were assumed for the analysis. For rich media i.e. YPD, the model was allowed to uptake all the trace elements, nitrogen sources, 20 amino acids, all the nucleotides and carbohydrates. Whereas in the minimal media, the model was allowed to uptake only glucose (carbon source), ammonium ions (nitrogen source) and trace elements.

### Reconstructing reported pathways in iPN730

In *L. kluyveri* (previously known as *S. kluyveri* in many studies) metabolic pathways have been reported with a particular focus on pyrimidine metabolism and overflow metabolism. URC pathway for pyrimidine degradation allowed the model iPN730 to grow on uracil, urea, and intermediates of the pathway as the sole nitrogen source. The pathway information on the URC pathway was obtained from the literature^48^. The metabolites and reactions for this pathway were incorporated into the model in COBRApy using reaction.add_metabolites() and model.add_reactions() respectively. URC6 gene locus encodes uracil phosphoribosyl transferase (EC: 2.4.2.9) which breaks down uracil to phosphoribosyl and 3-hydropropionate. URC3 and URC5 encode urea amidolyase (EC 3.5.1.54) which breaks down urea to ammonia and carbon dioxide. To check for the uptake of uracil and urea as a sole nitrogen source, the uptake for ammonium ion was restricted. The uptake rate for uracil and urea was set to 1000 mmol/gdw/h. The carbon source was kept as glucose while setting up an oxygen uptake rate very high for aerobic metabolism. FBA was performed to check for any in silico growth.

### Phenotypic phase plane analysis

Phenotypic phase plane analysis was conducted using doubleProductionEnvelop() function in COBRA Toolbox v3.0. The flux through transport reactions for ethyl acetate and ethanol i.e. EtAc_t and ‘etoh_t’ were used as a proxy for their production rates.

### Integration of gene expression data into iPN730 using GIMME algorithm

The gene expression data from previous study (GSE48135)^48^ from GEO Omnibus was used for constructing the context specific genome-scale metabolic model of *L. kluyveri*. We used GIMME (Gene Inactivity Moderated by Metabolism and Expression) algorithm^49^ for incorporating the gene expression data into the model. GIMME uses flux balance analysis along with gene expression data to prune reactions in the model according to their (1) gene expression and (2) contribution to metabolic objective. GIMME takes in the genome scale metabolic model, gene expression data and a user defined threshold for gene expression. GIMME tries to maximize the incorporation of reactions having expression of their constituent genes above the threshold while minimizing the reactions below it. We used a gene expression cutoff of 25th percentile as conducted in earlier studies^65,66^ with proven results.

The gene expression data (GSE48135) was normalized using custom scripts in R. The gene expression of *L. kluyveri* when growing on ammonium salt or uracil as the sole nitrogen source in minimal media was considered. We constrained the model to only uptake of either ammonium or uracil as nitrogen source prior to integration of gene expression. We used mapExpressionToReactions() to map the gene expression values to reactions based on their GPR relations. GIMME() function in COBRA Toolbox v3.0 was used for integration of gene expression into iPN730.

Flux Variability Analysis (FVA) was used for calculating the flux ranges of reactions of both the model. We used a loop-less version of FVA in COBRA Toolbox v3.0 to restrict the formation of futile cycles. The differential flux through reactions in each of the model was calculated using Jaccard index and the FVA ranges for both the models. Jaccard Index calculates the extent of intersection between given ranges. A Jaccard index of zero implies no overlap between two ranges.

Briefly, Jaccard index is calculated as follows:

For two given ranges/sets A and B,

$$ {\text{J}}\left( {{\text{A}},{\text{B}}} \right) = \frac{{\left| {{\text{ A }} \cap {\text{B }}} \right|}}{{\left| {{\text{ A }} \cup {\text{B }}} \right|}} $$

Using the minimum and maximum flux ranges obtained from FVA from both the models, we calculated the Jaccard Index for all the reactions. We considered all those reactions having a Jaccard index of zero between both the models as having differential flux. The differential reactions were then clustered based on their metabolic functionalities obtained from KEGG database.

## Supplementary information

Supplementary file 1

Supplementary file 2

## Data Availability

The metabolic model and associated data are supplied in the Supplementary Information. The associated codes are available in github (https://github.com/itsamit/Lachancea-kluyveri).
