# Supplementary material for: Reconstruction and analysis of genome-scale metabolic model of weak Crabtree positive yeast Lachancea kluyveri
Source: Sci Rep. 2020 Oct 1;10:16314. doi: 10.1038/s41598-020-73253-3 (PMC7530994; doi:10.1038/s41598-020-73253-3)
Supplement: Supplementary file 1 [file 41598_2020_73253_MOESM1_ESM.pdf]

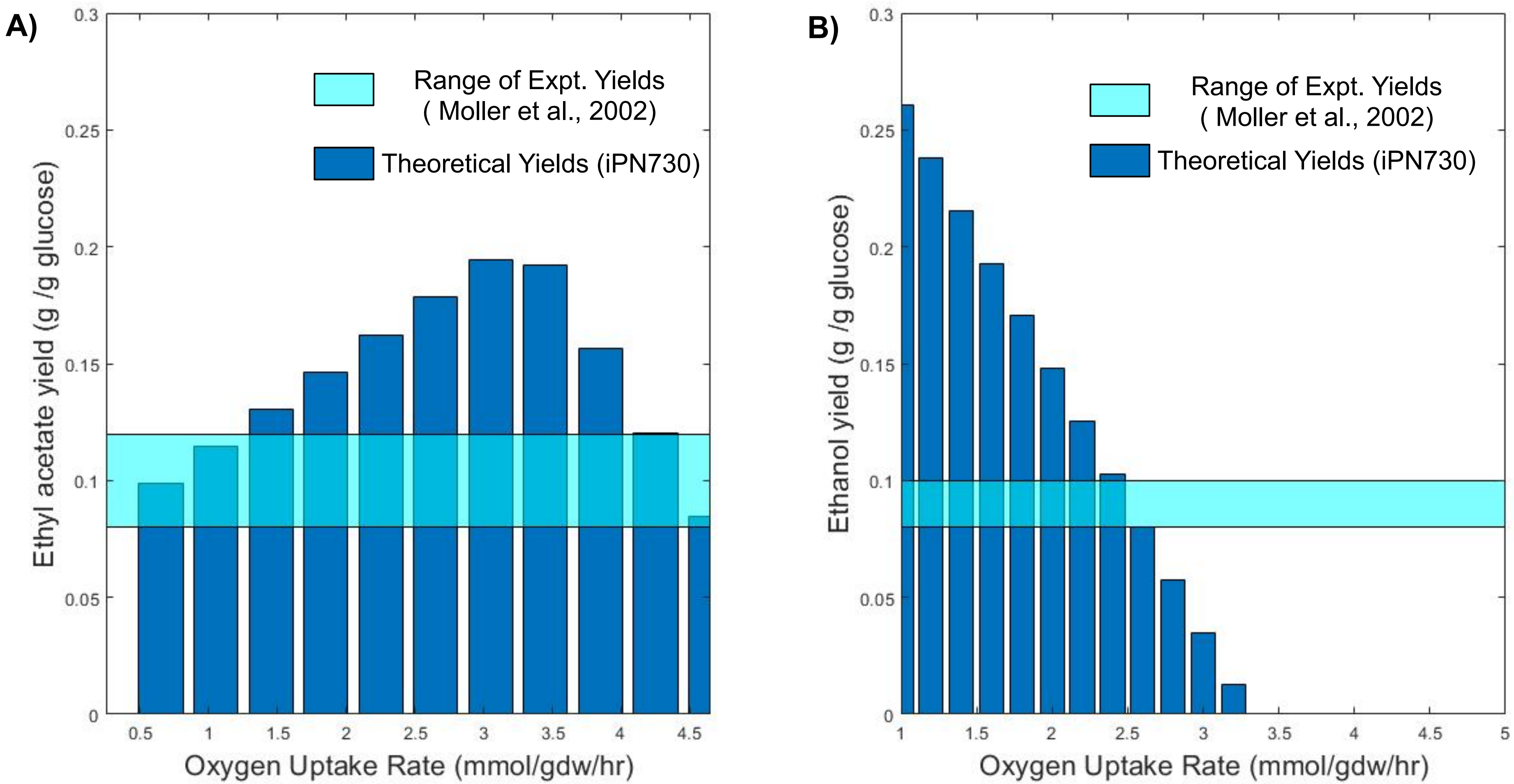

Figure S1. Comparison of yields from iPN730 using FBA with experimental yields for (A) ethyl acetate and (B) ethanol on glucose minimal media. The experimental range is demarcated in the horizontal bar.
